# Animal and Clinical Studies Evaluating Blood Glucose Control With Palatinose-Based Alternative Sweeteners

**DOI:** 10.3389/fnut.2020.00052

**Published:** 2020-04-28

**Authors:** Jaehyi Jang, Kyungae Jo, Ki-Bae Hong, Eun Young Jung

**Affiliations:** ^1^Department of Integrated Biomedical and Life Sciences, Graduate School, Korea University, Seoul, South Korea; ^2^BK21 Plus, College of Health Science, Korea University, Seoul, South Korea; ^3^Department of Food and Nutrition, Dongduk Women's University, Seoul, South Korea; ^4^Department of Home Economic Education, Jeonju University, Jeonju, South Korea

**Keywords:** palatinose, isomaltulose, alternative sweetener, blood glucose, glycaemic index

## Abstract

Animal and clinical studies were performed to determine whether Palatinose-based alternative sweeteners with increased sweetness contributed to blood glucose elevations. In the animal study, male Sprague-Dawley rats (6 weeks old) received an oral load of 2 g of glucose or Palatinose-based alternative sweeteners per kilogram of body weight. Thirty minutes after the glucose load, the rat blood glucose levels in the Palatinose-based alternative sweetener groups were significantly lower than those in the glucose groups (*p* < 0.05). Palatinose-based alternative sweeteners significantly improved glucose tolerance in rats. However, significant differences in the blood glucose levels were not observed among the Palatinose-based alternative sweeteners. In the clinical study, 14 healthy volunteers (21.4 ± 1.3 years) consumed glucose or Palatinose-based alternative sweeteners (50 g). At 60 min, when Palatinose-based alternative sweeteners were ingested, blood glucose was significantly lower compared to when glucose was ingested (Palatinose-L, 123.1 mg/dL; Palatinose-IS, 125.9 mg/dL; Palatinose-FOS. 129.1 mg/dL vs. glucose, 154.8 mg/dL, *p* < 0.05). The glycaemic index of Palatinose-L, Palatinose-IS and Palatinose-FOS was 43.9, 58.1, and 49.2, respectively. Palatinose-based alternative sweeteners could help maintain health as the postprandial blood glucose levels are constantly maintained owing to slow hydrolysis.

## Introduction

Palatinose is a disaccharide carbohydrate comprising glucose and fructose (chemical name: 6-*0*-α-D-glucopyranosyl-D-fructose) manufactured by the enzymatic rearrangement of sucrose from cane sugar. In 1980, Palatinose was first commercialized as a sweetener by the Japanese. Unlike xylitol and sorbitol, which are both sugar alcohols, Palatinose is completely hydrolysed by disaccharidase in the human small intestine, thus demonstrating no side effects such as flatulence or diarrhea after intake of large quantities (1, 2).

Palatinose tastes and appears similar to sucrose and is used as a sucrose substitute in most sweetened food products (3, 4). Furthermore, the energy supply from Palatinose attenuates blood glucose levels in the body (4). The hydrolysis and absorption of Palatinose is slow as reflected by the low blood glucose levels and the steady rise in the blood glucose response, corresponding with a low glycaemic index (GI) (5, 6). The oral intake of Palatinose results in significant improvements in diet-induced metabolic abnormalities, which could help prevent insulin resistance and its associated complications (5, 7).

Palatinose has a sweetness quality similar to sucrose, with a low sweetness intensity; although Palatinose is an isomer of sucrose, its sweetness intensity is only 42% that of sucrose. Additionally, Palatinose is a crystalline reducing disaccharide (8). Hence, it has low applicability owing to its low sweetness degree and high crystallinity. In this study, Palatinose-based alternative sweeteners were developed by partially converting some sucrose to compensate for the low sweetness and high crystallinity of Palatinose. Animal and clinical studies were performed to determine whether Palatinose-based alternative sweeteners with increased sweetness contribute to blood glucose level elevations.

## Materials and Methods

### Preparation of Palatinose-Based Alternative Sweeteners

In this study, Palatinose-L, Palatinose-IS, and Palatinose-FOS were provided by Neo Cremar Co. Ltd. (Seoul, South Korea). Briefly, sugar (400 g) was added to water (600 ml) and stirred at 75°C. After adjusting the pH of the aqueous sugar solution to 6, *Protaminobacter rubrum* CBS 574.77 (a primary enzyme) was added and allowed to react at 30°C for 20 h. After the enzymatic reaction was complete, the enzyme was inactivated by increasing the temperature to 90°C for 30 min. The treatment up to this stage generated Palatinose-L. Palatinose-IS was derived from invertase-treated Palatinose-L. Palatinose-FOS was prepared from Palatinose-L treated with fructosyltransferase. Both reactions were performed at 55–60°C for 20–24 h. The reactions were inactivated by increasing the temperature to 90°C for 30 min, followed by the addition of activated carbon and stirring at 65°C for 2 h. Subsequently, the solutions were filtered through a 0.5-μm filter, then passed through a 0.22-μm filter, and finally concentrated to 70°Brix. Table 1 in Supplementary Material shows the sugar composition of Palatinose-L, Palatinose-IS, and Palatinose-FOS.

### Animal Study

The study protocol was reviewed and approved by the Korea University Animal Care Committee, Korea (KUIACUC-2018-7). Six-week-old male Sprague-Dawley rats (312.67±4.32 g) were obtained from the Central Lab, Animal Inc. (Seoul, Korea). They were individually housed in plastic cages with stainless steel grate floors; the temperature was maintained at 24 ± 1°C, with 60% atmospheric humidity, and a 12-h light/dark cycle. After an adaptation period, the rats were categorized into four groups (six rats/group) based on blood glucose and body weight. Following an overnight fast, the rats received an oral load of 2 g of glucose or Palatinose-based alternative sweeteners (Palatinose-L, Palatinose-IS, or Palatinose-FOS) per kilogram of body weight. Glucose levels were measured at fasting and at 30, 60, 90, and 120 min using the glucose oxidase method in venous blood drawn from the tail vein. Blood samples were withdrawn onto test strips and analyzed using an Accutrend Sensor (Roche Diagnostics, Barcelona, Spain).

### Clinical Study

The clinical study protocol was approved by the Ethics Committee for Human Experimentation of the Jeonju University and was conducted in accordance with its rules and regulations (jjIRB-181115-BR-2018-1107). A within-subject and repeated-measures design was used. The clinical study was conducted in accordance with the procedure recommended by the Food and Agriculture Organization of the United Nations (FAO)/WHO (9). Individuals were excluded if they had diabetes; liver, gastrointestinal, or cardiovascular diseases; had a known hypersensitivity or allergy to Palatinose. On the day before testing, the subjects were asked to restrict their intake of alcohol and caffeine-containing drinks as well as avoid participating in any intense physical activity. Each subject participated in the experiment on four non-successive days. Each experimental day was spaced 1 week apart. On the first experimental day, the subjects consumed glucose (50 g). On the second experimental day, the subjects ingested Palatinose-L (50 g), on the third day, Palatinose-IS (50 g), and on the last day, Palatinose-FOS (50 g). Sweeteners were ingested within 3 min together with 200 ml of water. Glucose levels were measured at fasting and at 15, 30, 45, 60, 90, and 120 min using the glucose oxidase method in blood drawn from the fingertip. Blood samples were drawn onto test strips and analyzed using an Accutrend Sensor (Roche Diagnostics, Barcelona, Spain).

The GI was calculated in accordance with the procedure indicated by Kawai et al. (10) based on the incremental blood glucose area in relation to the corresponding area obtained after glucose was used as the reference food. The incremental area under the blood glucose response curve (IAUC) for each sweetener consumed by each subject was expressed as a percentage of the mean IAUC for glucose consumed by the same subject. The GI of each sweetener was assumed as the mean for the whole group.

### Statistical Analyses

All statistical analyses were performed using the Statistical Package for Social Sciences ver. 25.0 (SPSS, IL, USA). The differences in the results between sweeteners were statistically evaluated by one-way analysis of variance (ANOVA) and Tukey's multiple tests. All data were two-sided with a 5% significance level and are reported as the mean ± standard error of the mean (SEM).

## Results

**Figure 1:**
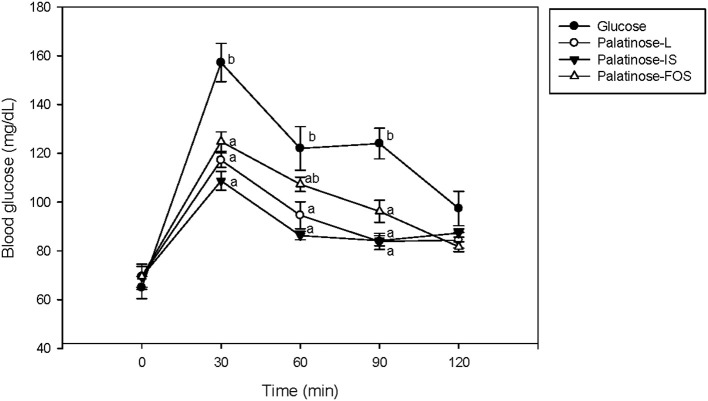
Changes in blood glucose levels in response to the oral administration of glucose or Palatinose-based alternative sweeteners (animal study). Values are the means ± standard error of the mean (SEM) for five rats. Means with different superscript letters are significantly different, with *p* < 0.05 according to Tukey's multiple range tests.

Figure 1 presents the changes in the levels of blood glucose following the oral administration of glucose or Palatinose-based alternative sweeteners in the animal study. The rat blood glucose levels observed in the Palatinose-based alternative sweetener groups were significantly lower than those in the glucose groups 30 min after the glucose load (*p* < 0.05). Palatinose-based alternative sweeteners significantly improved glucose tolerance in the rats. However, significant differences in blood glucose levels were not observed among the Palatinose-based alternative sweeteners. After 120 min, no significant differences were observed in the blood glucose levels between the glucose group and the Palatinose-based alternative sweetener groups.

**Figure 2:**
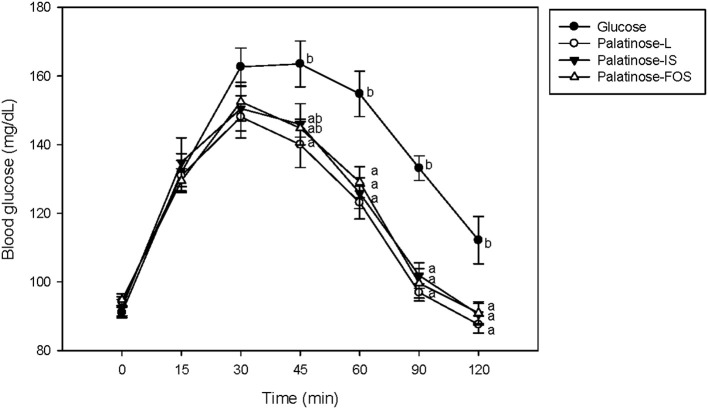
Changes in blood glucose levels in response to the oral intake of glucose or Palatinose-based alternative sweeteners (clinical study). Values are the means ± standard error of the mean (SEM) for 14 subjects. Means with different superscript letters are significantly different, with *p* < 0.05 according to Tukey's multiple range tests.

**Figure 3:**
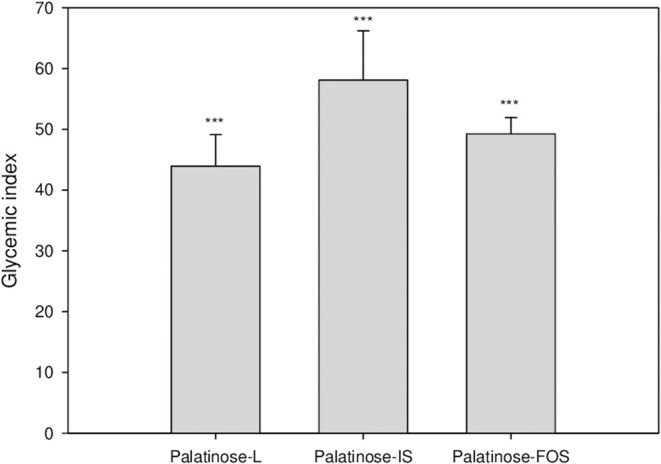
Glycaemic index following the oral intake of Palatinose-based alternative sweeteners. Values are the means ± standard error of the mean (SEM) for 14 subjects. The asterisk indicates a significant difference (*** *p* < 0.001) between glucose and Palatinose-based alternative sweetener by a repeated measure ANOVA followed by Bonferroni-adjusted pairwise comparisons within groups.

Figure 2 depicts the changes in the blood glucose levels in response to the oral intake of glucose or Palatinose-based alternative sweeteners in the clinical study. Fourteen healthy volunteers (female; 9, male; 5) aged 21.4 ± 1.3 years participated in this study. The subjects had mean body weights of 52.3 ± 2.2 kg and 74.67 ± 7.4 kg, height of 158.3 ± 1.1 m and 167.8 ± 2.3 m, respectively. The fasting concentrations of blood glucose were similar in all the groups. The blood glucose levels at 60 min after the oral intake of Palatinose-based alternative sweeteners (Palatinose-L, 123.1 ± 17.9 mg/dL; Palatinose-IS, 125.9 ± 16.8 mg/dL; Palatinose-FOS, 129.1 ± 16.8 mg/dL) were significantly (*p* < 0.05) lower than those after the oral intake of glucose (154.8 ± 4.6 mg/dL). After 60 min of the intake of glucose and Palatinose-based alternative sweeteners, the blood glucose levels were significantly (*p* < 0.05) different. After 120 min, the blood glucose levels in the Palatinose-based alternative sweetener groups returned to the levels observed in the fasting state; however, the blood glucose levels in glucose group remained elevated compared to those in the fasting state. The GI was calculated by the response curves of glucose; the results are shown in Figure 3. After 120 min, the IAUC for the Palatinose-based alternative sweeteners was significantly lower than that for glucose (*p* < 0.05). The GI for Palatinose-L (43.9 ± 19.5) than for Palatinose-IS (58.1 ± 30.2) or Palatinose-FOS (49.24 ± 10.1) were significantly lower compared to glucose (*p* < 0.001).

## Discussion

Palatinose is regarded as a healthier sweetener than sucrose for several reasons. Reportedly, orally ingested Palatinose minimally increases blood glucose and insulin levels and, additionally, suppresses the blood glucose elevations induced by other carbohydrates, such as glucose, sucrose, and dextrin (11–14). Several studies have reported that Palatinose is broken down at a slower rate than sucrose. Owing to its slow digestion, Palatinose reaches a more distal site in the human small intestine than sucrose as evidenced by their different incretin hormone responses. Compared with sucrose, the absorption of energy from Palatinose as a carbohydrate is prolonged (11–14).

Recent data support the preventive potential of a low GI diet against the development of type 2 diabetes and cardiovascular disease (15). In addition, the potential of low GI diets for body weight management has been reported. Several studies have shown that low GI foods (or lowering the GI of a food) reduces hunger and results in a lower energy intake (4, 7). The confirmation of the low GI response to Palatinose has been provided in numerous studies in different population groups, including healthy subjects, overweight or obese individuals, prediabetic patients, and patients with type 1 or type 2 diabetes (10, 16, 17). In these studies, all groups reported a lower blood glucose response to Palatinose, with an associated reduction in the blood insulin response. Palatinose, used in place of sucrose and many other sweeteners, has enabled the glycaemic profile of foods to be reduced. Moreover, several studies have documented that the regular consumption of Palatinose improves lipid metabolism compared to that with the consumption of other sweeteners (18–20).

Rats and humans receiving Palatinose-based alternative sweeteners demonstrated blood glucose levels and area under the curve results significantly different from those observed with glucose administration. In this study, the oral doses of Palatinose-based alternative sweeteners improved postprandial hyperglycaemia. Palatinose-L, Palatinose-IS, and Palatinose-FOS are expected to play a role as ideal sugar substitutes in line with the recent trend toward sugar reduction. These substitutes can be clarified as sweeteners suitable for individuals with diabetes due to their inherently low GI. Therefore, they showed the potential as an alternative sweetener to control blood sugar after eating.

## Data Availability Statement

All datasets generated for this study are included in the article/Supplementary Material.

## Ethics Statement

The clinical study was approved by the Ethics Review Board of Jeonju University, Korea (jjIRB-181115-BR-2018-1107). The patients/participants provided their written informed consent to participate in this study. The protocol for the animal study was reviewed and approved by the Korea University Animal Care Committee, Korea (KUIACUC-2018-7).

## Author Contributions

EJ made a substantial contribution to drafting the manuscript and interpreting and analyzing the data. KJ searched for study sites, prepared the manuscript, and conducted the study. KJ and K-BH designed the study, performed the statistical analysis, and responded to editorial and reviewers' comments. All authors read and approved the final manuscript.

## Conflict of Interest

The authors declare that the research was conducted in the absence of any commercial or financial relationships that could be construed as a potential conflict of interest.
