# Supplementary material for: Animal and Clinical Studies Evaluating Blood Glucose Control With Palatinose-Based Alternative Sweeteners
Source: Front Nutr. 2020 Apr 28;7:52. doi: 10.3389/fnut.2020.00052 (PMC7199504; doi:10.3389/fnut.2020.00052)
Supplement: Supplementary file 1 [file Table_1.docx]

**Table 1. Sugar composition of Palatinose-based alternative sweeteners**

| Sugar composition (%) | Palatinose-L | Palatinose-IS | Palatinose-FOS |
| --- | --- | --- | --- |
| Glucose | 1.0–3.5 | 35.0–40.0 | 18.0–21.0 |
| Fructose | 1.0–3.5 | 35.0–42.0 | 5.0–8.0 |
| Trehalulose | 0.5–2.0 | 0–2.0 | 0.5–2.0 |
| Palatinose | 20.0–22.0 | 0.5–2.0 | 20.0–22.0 |
| Sucrose | 70.0–78.5 | 20.0–22.0 | 18.0–26.0 |
| Fructooligosaccharides | - | - | 18.0–26.0 |
